# Supplementary material for: Production of human milk fat substitute by engineered strains of Yarrowia lipolytica
Source: Metab Eng Commun. 2022 Jan 6;14:e00192. doi: 10.1016/j.mec.2022.e00192 (PMC8752951; doi:10.1016/j.mec.2022.e00192)
Supplement: Multimedia component 1 [file mmc1.pdf]

Supplementary data.

Supplementary Figure 1. Codon optimised LPAT sequences use in this study.

```
>mLPAT1
AAGCTTATGTCCGACCTGTCTGGTGCTGCTACCCCCGAGTCCACCTACCCTGAGCCTGAGATCAAGCTGTCCTCTCGA
CTCCGAGGAATTTGCTTCTGTCTCGTCGCCGGCGTGTCTGCTATCGTCCTGATTGTGCTCATGATCACCGGCCACCCC
TTCGTCCTGCTCTTCGACCGATACCGACGAAAGTTCCACCACTTCATCGCCAAGCTGTGGGCTTCCATCTCTATCTAC
CCCTTCTACAAGACCGACATTCAGGGTCTGGAGAACCTCCCCTCCTCTGACACCCCCTGCGTCTACGTGTCCAACCAC
CAGTCTTTCCTGGACATCTACACCCTGCTCTCCCTCGGACAGTCTTACAAGTTCATTTCCAAGACCGGCATCTTCGTC
ATTCCCGTGATCGGCTGGGCCATGTCCATGATGGGTGTCGTGCCCCTGAAGCGAATGGACCCCCGATCTCAGGTCGAC
TGCCTGAAGCGATGTATGGAGCTCGTCAAGAAGGGTGCCTCCGTCTTCTTCTTCCCCGAGGGAACCCGATCTAAGGAC
GGACGACTGGGCCCCTTCAAGAAGGGCGCTTTCACCATTGCTGCTAAGACCGGTGTGCCTGTGGTGCCCATTACCCTG
ATGGGCACCGGCAAGATCATGCCCACCGGTTCCGAGGGAATTCTCAACCACGGTGACGTCCGAGTGATCATTCACAAG
CCCATCTACGGATCTAAGGCTGACCTGCTCTGTGACGAGGCCCGAAACAAGATTGCTGAGTCCATGAACCTGCTCTCT
TAACGATCGTTTTTTTTTATATATATATATATATATATATAACTGTCTAGAAATAAAGAGTATCATCTTTCAAAAAGC
TT

>AGPAT1
AAGCTTATGGACCTGTGGCCCGGAGCTTGGATGCTGCTCCTGCTCCTGTTCCTCCTCCTGCTGTTCCTCCTGCCCACC
CTGTGGTTCTGCTCCCCCTCTGCTAAGTACTTCTTCAAGATGGCCTTCTACAACGGTTGGATTCTGTTCCTGGCCGTC
CTGGCTATTCCCGTCTGTGCTGTGCGAGGACGAAACGTGGAGAACATGAAGATCCTCCGACTGATGCTCCTGCACATC
AAGTACCTGTACGGAATTCGAGTTGAGGTCCGAGGCGCCCACCACTTCCCTCCCTCCCAGCCTTACGTCGTGGTCTCT
AACCACCAGTCCTCTCTGGACCTCCTGGGTATGATGGAGGTGCTCCCTGGACGATGTGTCCCTATCGCTAAGCGAGAG
CTGCTCTGGGCTGGTTCCGCTGGACTGGCTTGTTGGCTGGCTGGCGTCATCTTCATTGACCGAAAGCGAACCGGTGAC
GCTATTTCCGTGATGTCTGAGGTGGCTCAGACCCTCCTGACCCAGGACGTTCGAGTCTGGGTGTTCCCTGAGGGAACC
CGAAACCACAACGGTTCCATGCTGCCCTTCAAGCGAGGCGCCTTCCACCTCGCTGTCCAGGCTCAGGTCCCTATTGTG
CCCATTGTCATGTCCTCTTACCAGGACTTCTACTGCAAGAAGGAGCGACGATTCACCTCTGGACAGTGTCAGGTCCGA
GTGCTGCCTCCCGTGCCTACCGAGGGACTGACCCCCGACGACGTTCCTGCTCTGGCTGACCGAGTCCGACACTCCATG
CTGACCGTGTTCCGAGAGATTTCTACCGACGGTCGAGGCGGTGGAGACTACCTCAAGAAGCCCGGCGGTGGAGGCTAA
CGATCGTTTTTTTTTATATATATATATATATATATATATAACTGTCTAGAAATAAAGAGTATCATCTTTCAAAAAGCTT

>LPAAT2
AAGCTTATGTCTGTCCTCACCAAGTGGCTGGGTCTCCCCTCTTTCCTGTTCTCCGTCTTCGTGTTCTACTGGTCTCTC
CCCATCTTCGCCATTCTGTACCGAATCCGATTCGCTTCCCTGGGAAAGCGAAACGACATGCTCGACTGGGCTCGAGCC
CTGGTCGCCTACTTCCGAGTGACCCTGCTCCAGGCTGGCGAGCACACCCTGTACAAGGGCGGTCCCTGCCTGTACCTC
TGTAACCACCGATCCTGGGCTGACTTCTTCATTGACGCTTACCTGACCGAGGGACGAGCTGCTCTCATGTCTCGATGG
CTGGTCTACTTCGTGTTCCCCGTCTTCTGCACCTCCTGTATGATCCTCAAGGGTATTGTCCTGTTCAAGCGAGGAACC
ATTGCCGACAAGGAAGCCTTCAACGCCTGGCTGGACCAGACCCTGGGATCCTCTCACGTCCCTGGACTGCTGGTGTAC
CCCGAGGGACACCGATCTACCAAGCCTGCCTCCCTGCCTCTCAAGCGAGGTATGCTCCACTACGCTCACTCTCGAAAG
CTGCCCGTGCAGATTGTCGTGACCCGAGGCAAGGACGAGGTCCTGTCCGAGAAGTCTCAGTCCGTGCACTTCGGACGA
ACCTGCGTCACCACCTTCTCTAAGGTGCTCAAGTCCGCTGACTACCCCAACTTCGAGGCCTTCTTCACCGACCTGCAG
GCTACCTGGGACTCTTGTTGGGCCGCTACCTACGGACTGGAGGACCTCAAGAACGTGCCTCGATTCTCTATGCCCGGA
CCTCAGGCCTACTCCTACTCCTCTTCCATGTGGGTGCAGCAGCTCGCCATCACCCTCGTGTCTATTCTGGTCTTCGCT
GGAGTTTGTTACGGCTCCTGGCGAGGTCTGGCCGCTGCCCTGGCTGCTACCGGTGCTGCCCAGCAGGTGGTTGCTCTG
GTGCTGGCTGCTTGGGTGGGTTCTTCCGTGCTCCGATCCTTCCTGTAACGATCGTTTTTTTTTATATATATATATATA
TATATATAACTGTCTAGAAATAAAGAGTATCATCTTTCAAAAAGCTT
```

Supplementary Table 1. Plasmids and PCR primers used in this study.

Plasmid	Genotype	Source/description
pFA6aURA3-09	*Parent Plasmid (5'_MCS-loxP-PPURA3TT-loxP-MCS_3')*	Bhutada et al., (2017)
pGMKGSY12	*YlGSY1*^{P}*-loxP-PURA3T-loxP-YlGSY1*^{T}	Bhutada et al., (2017)
YlmLPAT1	*pUC57-5'_mLPAT1-Syn*^{T}*_3'*	This work
YlAGPAT1	*pUC57-5'_AGPAT1-Syn*^{T}*_3'*	This work
YlLPAAT2	*pUC57-5'_LPAAT2-Syn*^{T}*_3'*	This work
pGSYTEF	*YlGSY1*^{P}*-TEF1*^{P}*-loxP-PURA3T-loxP-YlGSY1*^{T}	This work
pTEF-mLPAT1	*YlGSY1*^{P}*-TEF1*^{P}*-mLPAT1-Syn*^{T}*-loxP-PURA3T-loxP-YlGSY1*^{T}	This work
pTEF-AGPAT1	*YlGSY1*^{P}*-TEF1*^{P}*-AGPAT1-Syn*^{T}*-loxP-PURA3T-loxP-YlGSY1*^{T}	This work
pTEF-LPAAT2	*YlGSY1*^{P}*-TEF1*^{P}*-LPAAT2-Syn*^{T}*-loxP-PURA3T-loxP-YlGSY1*^{T}	This work

Primers	Sequence
TEF-GSY-F	CTCGCAACAACCGATTCCAACAAGAGACCG GGTTGGCGGCGCA
TEF-GSY-R	ATAACTTCGTATAATGTATGCTATACGAAGT TATAAGCTTTGAATGATTCTTATACTCAGAA GGAAATGCTTAA
GSY1^{P}*-F*	GAGGAGCTGTTGGAGGTACGC
GSY1^{T}*-R*	GAACATGTGTGCGTTTTCACTTTCG

mLPAT1_R	GTTCTCCAGACCCTGAATGTCG
AGPAT1-R	GACCTCAACTCGAATTCCGTAC
LPAAT2-R	GCCATCGAGACATGAGAGCAGC

Supplementary Table 2. Total and sn-2 FA composition of TAG from *Y. lipolytica* strains cultured on glycerol in nitrogen-limited media. Values are means ±SD of measurements made on three separate cultures for each genotype.

Strain	16:0	16:1	18:0	18:1	18:2
Total FA composition of TAG (%)					
WT	21.3 ±1.2	6.5 ±0.5	11.7 ±0.4	46.3 ±2.4	14.2 ±1.2
gsy1Δ	9.7 ±0.9	4.7 ±0.6	11.5 ±0.8	58.0 ±2.2	16.1 ±1.4
gsy1Δ-mLPAT1	9.0 ±0.2	3.7 ±0.1	14.2 ±0.1	60.5 ±0.5	12.7 ±0.2
gsy1Δ-AGPAT1	16.6 ±0.6	3.6 ±0.3	11.3 ±0.3	52.8 ±0.8	15.7 ±0.5
gsy1Δ-LPAAT2	19.2 ±1.3	3.3 ±0.2	12.6 ±4.1	50.3 ±4.2	14.6 ±1.8
FA composition at the sn-2 position of TAG (%)					
WT	2.2 ±1.3	2.1 ±0.2	1.9 ±1.9	73.1 ±0.5	20.7± 2.4
gsy1Δ	1.6 ±0.5	1.2 ±0.5	0.6 ±0.6	75.6 ±1.5	21.0 ±1.7
gsy1Δ-mLPAT1	3.1 ±0.5	1.1 ±0.1	1.6 ±0.4	77.2 ±1.3	17.0 ±0.3
gsy1Δ-AGPAT1	28.7 ±0.5	3.2 ±0.1	1.4 ±0.2	49.0 ±0.4	17.7 ±0.8
gsy1Δ-LPAAT2	35.9 ±1.4	3.3 ±0.8	3.6 ±3.8	43.7 ±1.7	13.4 ±1.2

Supplementary Table 3. Total FA composition of palm oil use as a substrate in this study.

FA	14:0	16:0	18:0	16:1	18:1	18:2	18:3
%	0.7 ±<0.1	37.3 ±0.5	4.2 ±0.2	nd	35.5 ±1.0	10.4 ±0.4	0.6 ±<0.1

Values are means ±SD of measurements made on three samples. nd is not detected.

Supplementary Table 4. Cell biomass and lipid content of the *Y. lipolytica obese-gsy1Δ-LPAAT2* strain batch cultured in a bioreactor on glucose + palm oil in nitrogen-limited media for up to 96 h. The values are means ±SD of measurements made on three separate cultures.

	24h	48h	72h	96h
Cell biomass (g L^{-1})	3.9 ±0.3	4.6 ±0.8	5.5 ±0.5	5.0 ±0.6
Lipid content (% of CDW)	53.3 ±3.0	58.1 ±0.8	63.2 ±1.1	67.0 ±2.3

Supplementary Table 5. FA composition of TAG in *Y. lipolytica obese-gsy1Δ-LPAAT2* strain batch cultured in a bioreactor on glucose + palm oil in nitrogen-limited media for 72 h. The values are means ±SD of measurements made on three separate cultures.

FA	16:0	16:1	18:0	18:1	18:2
%	26.7 ±0.6	0.9 ±0.1	1.3 ±0.1	44.1 ±0.2	25.5 ±0.7
